# A Simple Model System Enabling Human CD34^+^ Cells to Undertake Differentiation Towards T Cells

**DOI:** 10.1371/journal.pone.0069572

**Published:** 2013-07-23

**Authors:** Antonio Lapenna, Christopher B-Lynch, Chrysa Kapeni, Richard Aspinall

**Affiliations:** 1 Regenerative Medicine Group, Cranfield Health, Cranfield University, Cranfield, United Kingdom; 2 Department of Immunology and Cancer Research, Faculty of Medicine, the Hebrew University of Jerusalem, Jerusalem, Israel; French Blood Institute, France

## Abstract

**Background:**

Channelling the development of haematopoietic progenitor cells into T lymphocytes is dependent upon a series of extrinsic prompts whose temporal and spatial sequence is critical for a productive outcome. Simple models of human progenitor cells development depend in the main on the use of xenogeneic systems which may provide some limitations to development.

**Methods and Findings:**

Here we provide evidence that a simple model system which utilises both human keratinocyte and fibroblast cell lines arrayed on a synthetic tantalum coated matrix provides a permissive environment for the development of human CD34⁺ haematopoietic cells into mature CD4⁺ or CD8⁺ T lymphocytes in the presence of Interleukin 7 (IL-7), Interleukin 15 (IL-15) and the Fms-like tyrosine kinase 3 ligand (Flt-3L). This system was used to compare the ability of CD34^+^ cells to produce mature thymocytes and showed that whilst these cells derived from cord blood were able to productively differentiate into thymocytes the system was not permissive for the development of CD34^+^ cells from adult peripheral blood.

**Conclusions/Significance:**

Our study provides direct evidence for the capacity of human cord blood CD34^+^ cells to differentiate along the T lineage in a simple human model system. Productive commitment of the CD34⁺ cells to generate T cells was found to be dependent on a three-dimensional matrix which induced the up-regulation of the Notch delta-like ligand 4 (Dll-4) by epithelial cells.

## Introduction

The generation of T cells from haematopoietic progenitor cells requires the positioning of progenitors within the thymus where a unique environment induces supports and directs their differentiation [1]. Production of new thymocytes continues throughout life and because the progenitors cannot be stored and maintained indefinitely within the thymus, continuation of production requires seeding of the thymus with these cells. Analysis of thymic output reveal that the rate of production of new T cells declines with age [2] and that as thymocyte production decreases so there is atrophy of the thymus.

In broad terms thymic atrophy has been linked to deficits in the progenitors seeding the thymus or to lesions in the environment provided by the thymic stromal cells. Studies utilising mouse systems have revealed that neither of these are mutually exclusive with experiments on both aspects aided by the use of surgical techniques, fetal thymic organ culture (FTOC) systems or allogeneic cell lines such as mouse bone marrow-derived OP9 cells expressing the Notch delta-like ligand 1 (OP9-Dll1) [3–5]. But the experiments in human systems have proved more intractable. Analysis of the capacity of haematopoietic progenitor cell populations to produce T cells have proceeded but has been hampered, mainly through the use of xenogeneic model systems which by their very nature are limited and associated with incomplete or inefficient differentiation of the progenitors [5]. Some studies of thymic stromal cells have indicated changes with age in the thymic environment cell type composition and expression profile but these data were limited by the lack of culture methods which could effectively model the thymic architecture in vitro [6].

With this in mind we developed a synthetic biology approach to the problem combining the use of freely available cell lines, engineered materials and suitable biochemical factors to induce human thymopoesis in vitro. Our aim was to induce differentiation along the T cell lineage using a simple model system containing only cells of human origin. To reach this aim we took inspiration from a recent study which showed how a human thymic microenvironment could be engineered using skin derived fibroblast and epithelial cells. Within this environment bone marrow derived CD133⁺ haematopoietic progenitor cells could be triggered to differentiate into T lymphocytes [7]. Unfortunately this work had problems. Derivation of cells from the skin lead to the possible contamination of the T cells derived from the bone marrow stem cells with those transported into the system through their sequestration within the stromal cells from human biopsies so that skin resident T lymphocytes amplification may have occurred [8]. A second problem arose when others found these results difficult to replicate [9].

To overcome these problems we constructed a three-dimensional thymus by attaching human keratinocytes and fibroblasts from cell lines to a tantalum coated matrix and then we seeded these cultures with CD34^+^ cells derived either form cord blood or from adult blood. Interestingly, differentiation of these cells along the T cell lineage occurred only with cord blood derived CD34^+^ cells. Moreover we analysed the biological characteristics of the artificial construct and this enabled us to hypothesize why providing a three-dimensional cellular architecture is essential to recreate the unique functions and characteristics of the thymic environment in vitro.

## Materials and Methods

### Ethics statement

Cord blood samples were collected from consenting mothers following birth and adult blood by venepuncture from a 55 years old adult donor following ethical permission by The Royal Marsden Local research Ethics Committee. The participants provided written informed consent.

### CD34^+^ cell separation

Mononuclear cells were separated from whole blood by gradient centrifugation using Ficoll-Paque (GE Heatlhcare) and subsequently depleted of CD2 and CD20 cells and enriched for CD34 using Microbeads (Miltenyi) according to MACS method on a VarioMACS magnet [10]. The separated cells were analyzed by flow cytometry and consisted of a unique highly pure CD34^+^CD45^lo^ population. Purity (considered as CD34 expression out of total CD45) was always > 90% in all separations, and viability, evaluated by staining the cells with 250 ng/ml Propidium Iodide solution (Sigma Aldricht), always >99%, No CD3 nor CD20 contaminating lymphocytes were detected. These freshly separated and collected CD34^+^cells were then used for the T cell differentiation studies.

### Culture of keratinocytes and fibroblasts

The HaCaT cell line (CLS, DKFZ) [11] were cultured in DMEM medium (Sigma-Aldrich) supplemented with 10% heat-inactivated fetal bovine serum (FBS), 2mM L-glutamine and 10% antibiotics (penicillin 100U/ml and streptomycin 100mg/ml) at 37^°^C with 5% CO_2_. The cells were passaged when less than 80% confluence. Primary Fibroblasts were purchased from Invitrogen, and cultured in Medium 106 (Invitrogen) supplemented with 2% heat-inactivated fetal bovine serum (FBS), hydrocortisone 1mg/ml, human-Epithelial Growth Factor 10ng/ml, human-basic Fibroblasts Growth Factor 3ng/ml, heparin 10µg/ml and 1X gentamycin/amphotericin. The fibroblasts were used at less than 15 passages. Three-dimensional skin constructs were performed on 9-mm × 9-mm × 1.5-mm tantalum coated carbon Cellfoam matrices (Cytomatrix) incubated with 100 µg/ml rat tail collagen I (Sigma Aldricht) and seeded with 1x10^5^ HaCaT Keratinocytes and 5×10^4^ primary fibroblasts, in culture medium consisting of a 1:1 mixture of the fibroblast and keratinocyte media described above. After 5 hours at 37^°^C, 5% CO_2_, matrices were moved to a new 24-well plate and 2 ml of a 1:1 mixture of the two media described above was added. The skin cell constructs were cultured for 6 days and medium was changed every other day. On day 6, CD34⁺ cells, isolated from either umbilical cord or adult peripheral blood, were added to each matrix, and the unit cultured in DMEM supplemented with 10% heat-inactivated FCS (Sigma-Aldrich), 20 ng/ml IL-7 (Miltenyi), 20 ng/ml IL-15 (Miltenyi), 100 ng/ml Flt-3L ligand (Miltenyi) and penicillin/streptomycin (Sigma-Aldrich). One-half of the medium was aspirated and replaced every 3 days, and the coculture maintained for up to 3-4 weeks.

### Flow Cytometry Analysis

Cell suspensions were analyzed using different combinations of conjugated monoclonal antibodies (mAbs) and their corresponding isotype controls after pre-incubation for 10 minutes at 4^o^C with 10 µl of FcR blocking reagent (Miltenyi). All antibodies were obtained from BD Biosciences unless stated otherwise, and were used according to the manufacturer’s instructions. The following mAbs (clones) were used: CD1a (HI149), CD3 (UCHT1), CD4 (RPA-T4), CD45 (HI-30), CD8 (SK-1), CD7 (6B7), CD38 (HIT-2), CD10 (HI-10), HLA-DR (G46-6), CD11c (Biolegend 3.9), CD56 (Biolegend MEM-188), CD135-APC (Biolegend BV 10A4H2), CD45/ CD34 cocktail (Miltenyi MB4-6D6/AC136), CD20 (Miltenyi LT20), Analysis of flow cytometry samples was performed on a C6 Accuri instrument.

### Reverse transcriptase-polymerase chain reaction

The RNA was isolated using Trizol (Invitrogen) and total RNA (1 µg) in 20 µl was transcribed into cDNA using the high capacity cDNA Reverse Transcription kit (Applied Biosystems). The cDNA product was mixed with QIAGEN SYBR Green Reagent and primers, and Real-time PCR performed using a CFX96 Bio-Rad real time PCR system (Bio-Rad). For the generation of standard curves, gene inserts were amplified using Green GoTaq Flexi DNA Polymerase (Promega), and the PCR product size controlled by 1.5% agarose gel electrophoresis. DNA concentration was measured with a spectrophotometer (Picodrop) and serial dilutions prepared starting from 10^11^ copies/µl as calculated by using Avogadro’s formula. All cDNA samples were normalized to ribosomal protein subunit 29 (RPS-29) housekeeping gene signals [12]. Primers used were as follows (anneal temperature): Dll-1 forward 5’ CTGATGACCTCGCAACAGAA3’ reverse 5’ ATGCTGCTCATCACATCCAG3’ (60^°^C), Dll-4 forward 5’-ACTGCCCTTCAATATTCACCT-3’ reverse 5’ GCTGGTTTGCTCATCCAATAA3’ (60^°^C), IL-7 forward 5’ TGAAACTGCAGTCGCGGCGT3’ reverse 5’ AACATGGTCTGCGGGAGGCG3’ (57^°^C), RPS-29 forward 5’ GCTGTACTGGAGCCACCCGC3’ reverse 5’ TCCTTCGCGTACTGACGGAAACAC3’ (55-60^°^C). 

### Western immunoblotting

Cells were lysed on ice in a buffer containing 150 mM NaCl; 50 mM Tris, pH 7.5; 1% NP-40 (Fisher Bioreagents), and total proteins re-solubilised in 1% TBS-Tween (Acros Organics) supplemented with complete protease inhibitor mixture (Roche Applied Science). Protein concentration was determined in all cell extracts using a Micro BCA Protein Assay Kit (Thermo Scientific). The samples (30 µg proteins) were electrophoresed on a SDS-PAGE gel (Invitrogen) and electrotransferred to a polyvinylidene difluoride membrane (Millipore) using an electro-blotter (Bio-Rad). The membrane was then incubated with primary antibodies overnight and protein bands detected with infra-red labelled secondary antibodies on the Odyssey Infrared Imaging System (LI-COR). Dll-4 protein was detected with 1:500 rat polyclonal anti-human Dll-4 (Enzo Life) plus 1: 10000 goat anti-rat IgG IRDye 800 (LI-COR) and normalized to β-actin using 1:10000 mouse IgG2a isotype anti-human-β-actin (Sigma-Aldrich) plus 1:10000 goat anti-mouse IgG IRDye 680 (LI-COR).

### TREC analysis

DNA was isolated from blood and newly generated CD3^+^ cells using Trizol reagent (Invitrogen) according to the manufacturer’s instructions and DJ signal join–type T-cell receptor excision circles (sj-TREC) were assayed. DNA (50 ng) was used in each RPS-29, sj-TREC PCR reactions in order to calculate TREC: T cell ratios as previously described [2]. Separated CD34^+^ cells were also analyzed in order to exclude T cell contamination.

### Statistics

All comparisons were assessed using Student *t* test. Results were considered significant if the *P* value was less than 0.05.

## Results

### De novo generation of cells in three-dimensional matrices

**Figure 1:**
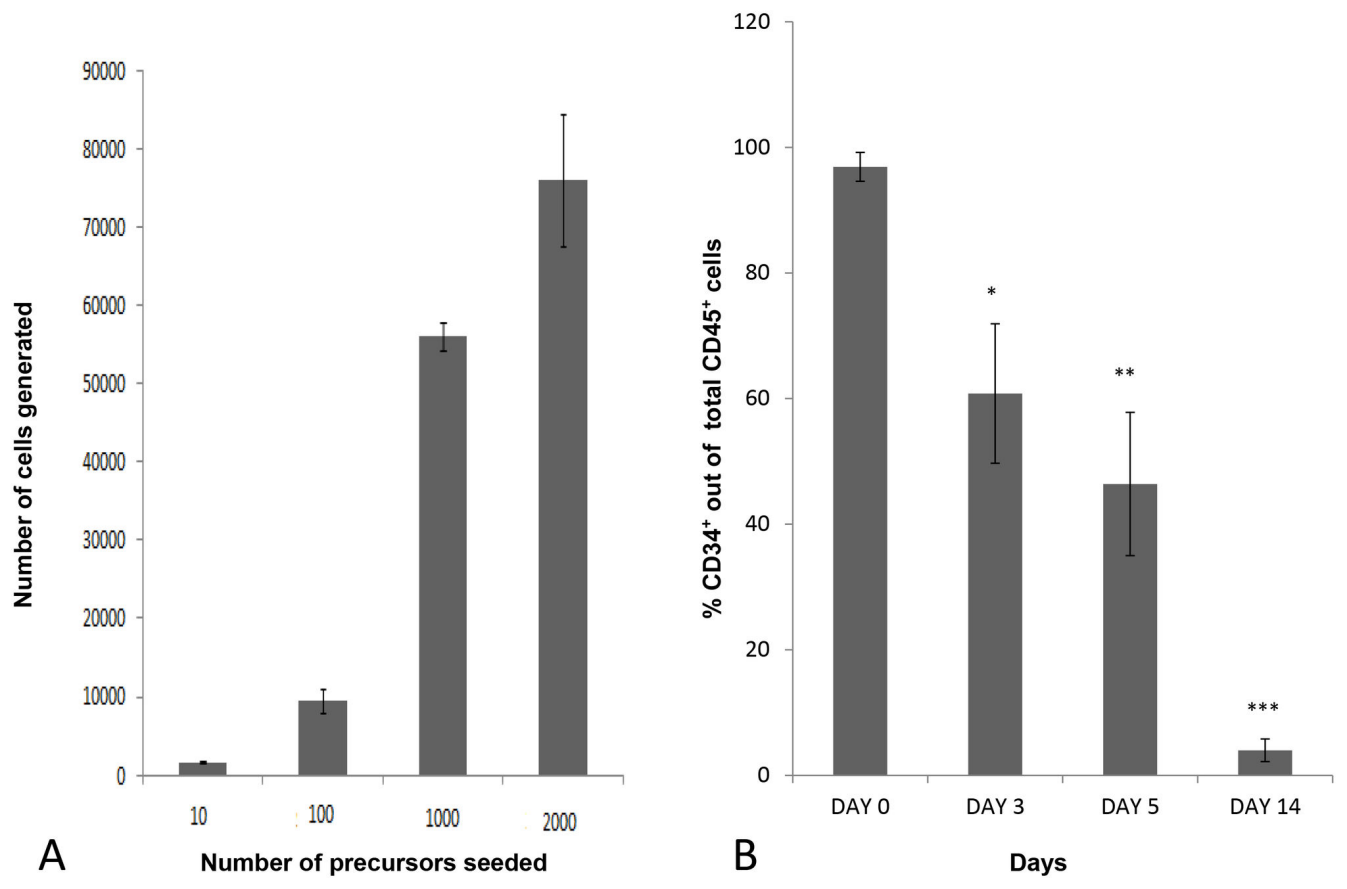
Expansion and differentiation of CD34^+^ cells. (A) Correlation between the initial number of CD34^+^ cells seeded and the amount of mature cells generated at day 14^th^. The results are the average ± standard derivation of three different experiments. (B) Progressive decline with time of CD34 expression among cord blood cellscultured in the matrix. The results are the average of three different experiments ± standard derivation. The differences between the 3^rd,^ 5^th^ and 14^th^ day and the seeded population are all significant (*p< 0.001; **p< 0.001; ***p< 0.001).

**Figure 2:**
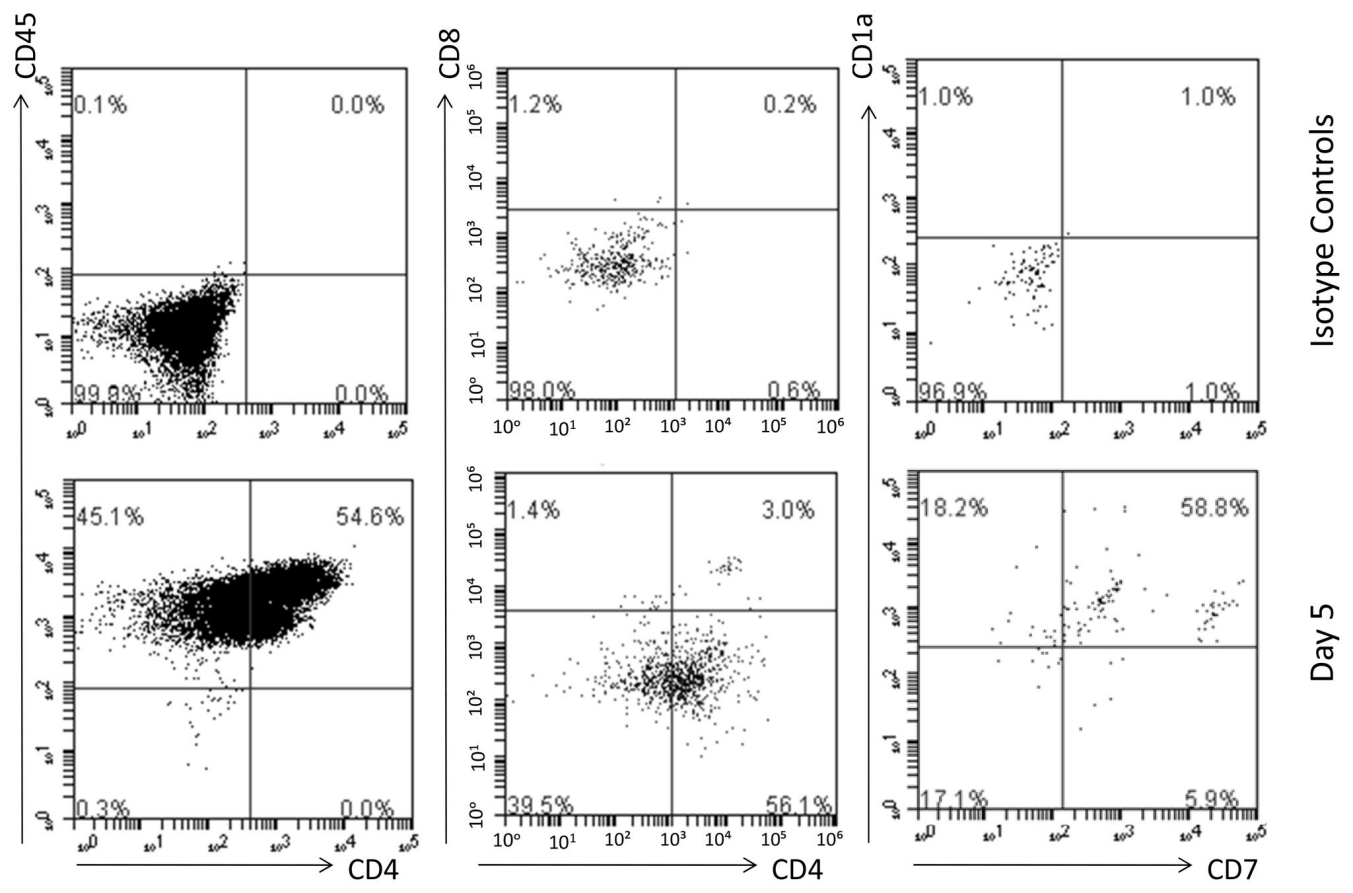
Kinetics of thymocytes generation. (A) By day 5 CD4 ^dim^intermediate single positive and some double positive CD4^+^CD8^+^ cells were present. These progenitors all expressed CD45, either high or dim and analysis of cultures also showed the presence of CD1a^+^CD7^+^ and CD1a^+^CD7 cells The images are representative of three different experiments.

**Figure 3:**
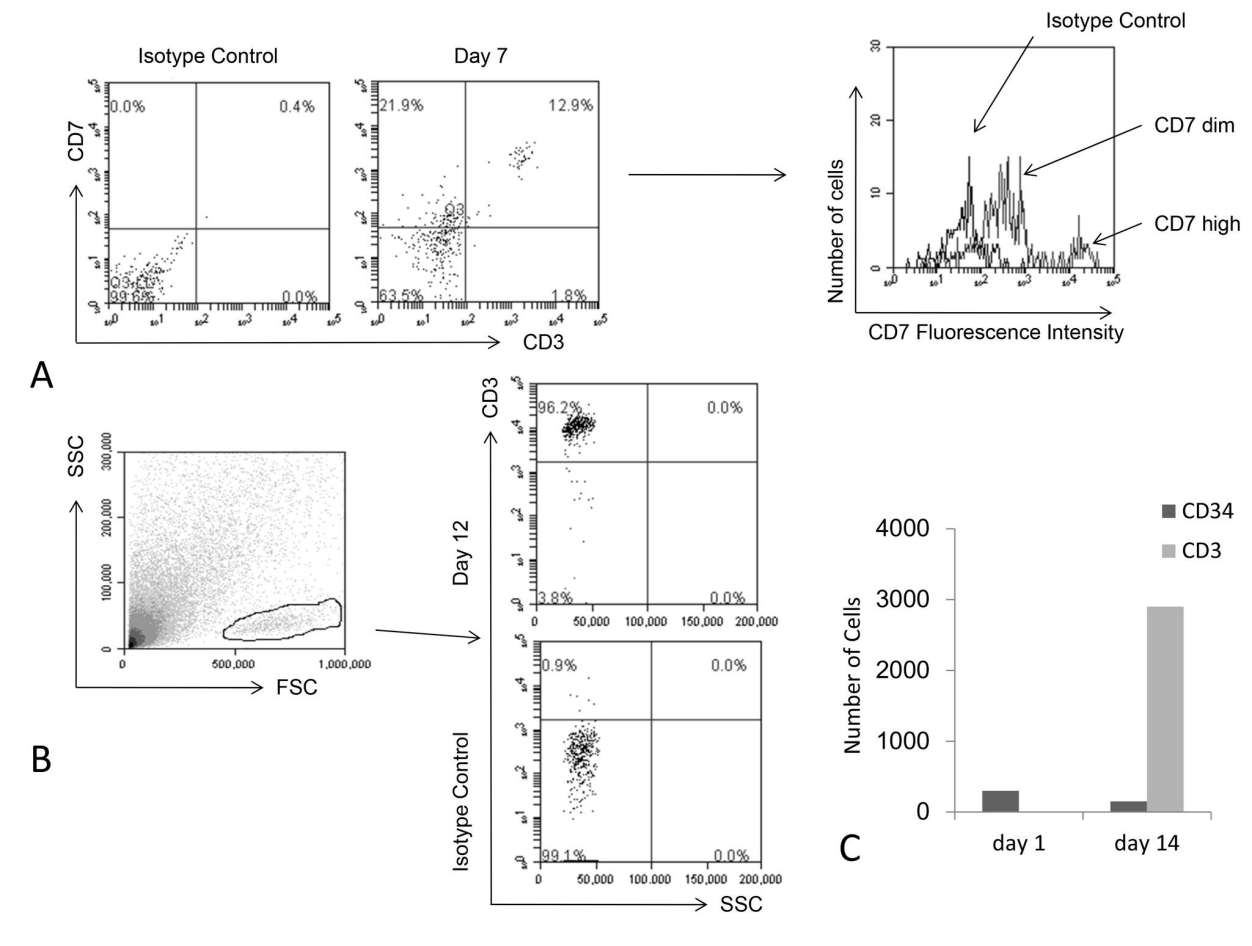
Generation of CD3+ thymocytes. (A) CD7^hi^CD3^hi^ and CD7 ^dim^ CD3⁻ cells were detected at day 7. (B) By day 12 approximately 90% of all the cells generated were CD3^+^ thymocytes. (C) A matrix seeded with approximately 300 CD34^+^ cord blood derived progenitors generated about 2900 CD3^+^ cells after 14 days. At that time about 150 CD34^+^ progenitors were still present whereas no other cell types were detected. The image A is representative of three different experiments while images B and C show a single experiment.

**Figure 4:**
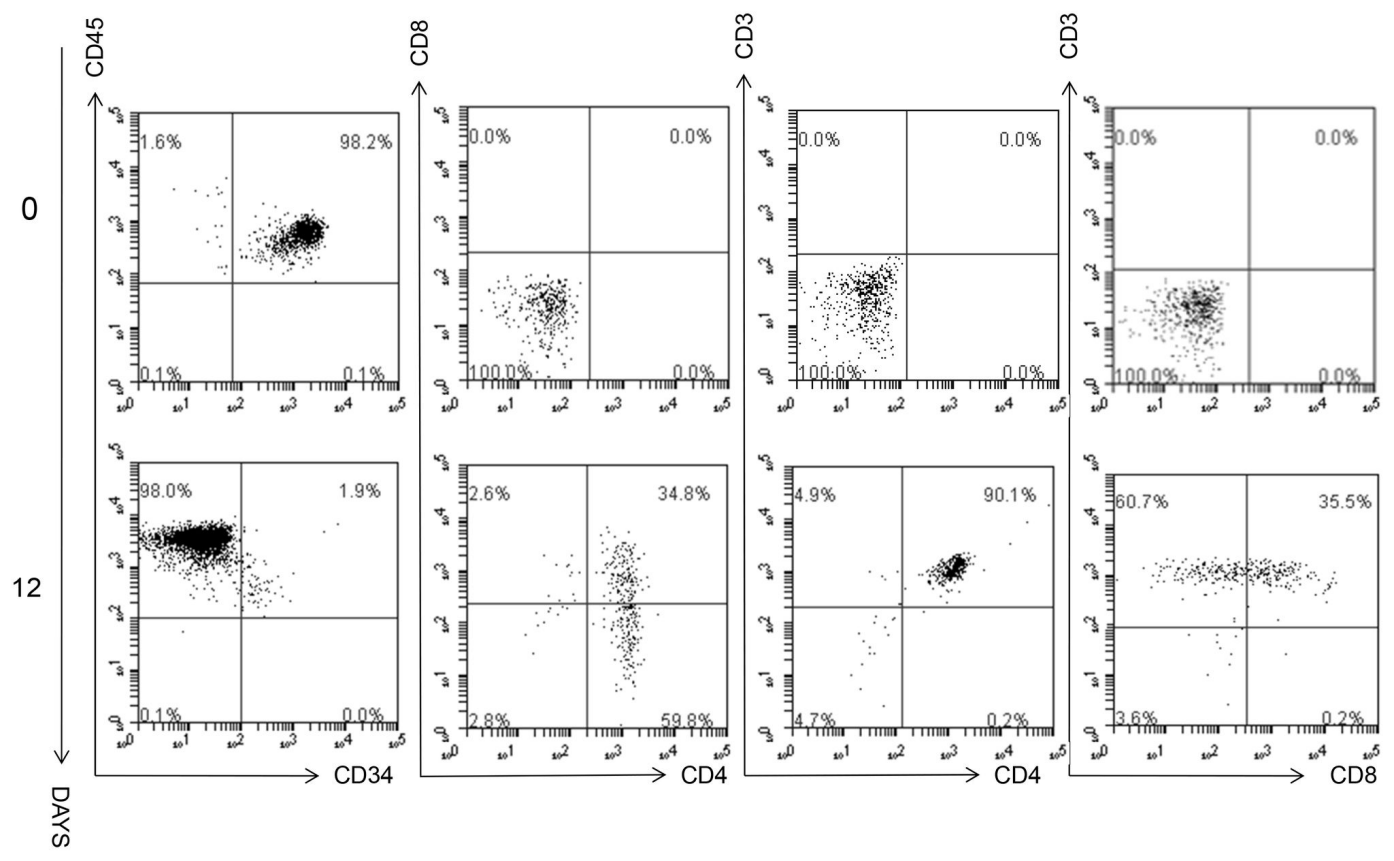
Most of generated cells are mature thymocytes by day12. The presence of double positive CD4^+^CD8^+^ and either CD4^+^ or CD8^+^ single positive CD3^+^ thymocytes was evident by day 12 when only about 2% of total CD45^+^ cells still expressed CD34. The images are representative of three different experiments.

Initial experiments with serial dilutions of cord blood CD34^+^ cells showed an exponential correlation between the initial number of CD34⁺ cells seeded and the total amount of CD45⁺CD34⁻ mature blood cells collected from the supernatant of cell-coated matrices at day 14 (Figure 1A). Cells were regularly shed into the supernatant and these were collected whilst feeding the cultures at 2-3 day intervals and analysed. The cells in the supernatant all expressed CD34 initially but this abruptly and progressively declined with culture time (Figure 1B) whilst an increasing percentage of the population started to express CD7, CD38 and CD1a. By 5 days we were able to detect CD4 ^dim^ISP and also able to identify a small number of DP CD4^+^CD8^+^ cells, all cells expressed CD45 and analysis of cultures also showed the presence of cells which were CD1a^+^CD7^+^ or CD1a^+^CD7^-^ cells (Figure 2). The first few CD3^+^ cells appeared at 7 days as CD7^hi^ cells (Figure 3A), and later CD3 expression further increased. In one experiment matrices were seeded with approximately 300 CD34⁺ cells and analysis at day 12 revealed that more than 90% of the cells generated were CD3^+^ (Figure 3B). On day 14 we were able to collect about 2900 CD3^+^ cells (Figure 3C). At 12 days CD3^+^CD4⁺CD8⁺, CD4⁺CD3^+^ and CD8⁺CD3^+^ cells were present, only few CD45^+^ cells still expressed CD34 and most of the CD3^+^ cells were CD4^+^CD8^-^ (Figure 4).

This phenotypic data revealed that within this model system differentiation was ordered and progressed along the normal developmental pathway as judged by the sequential appearance of the expected intermediate stages in the production of mature thymocytes [13–15].

CD34^+^ cells seeded onto monolayer cultures of fibroblasts and keratinocytes seeded at the same ratio failed to differentiate and died within 3 days. These cultures were provided with the same cocktail of growth factors as the three dimensional cultures which indicated significant changes in the keratinocytes and fibroblasts induced by this conformational change.

### Analysis of the three-dimensional matrix

**Figure 5:**
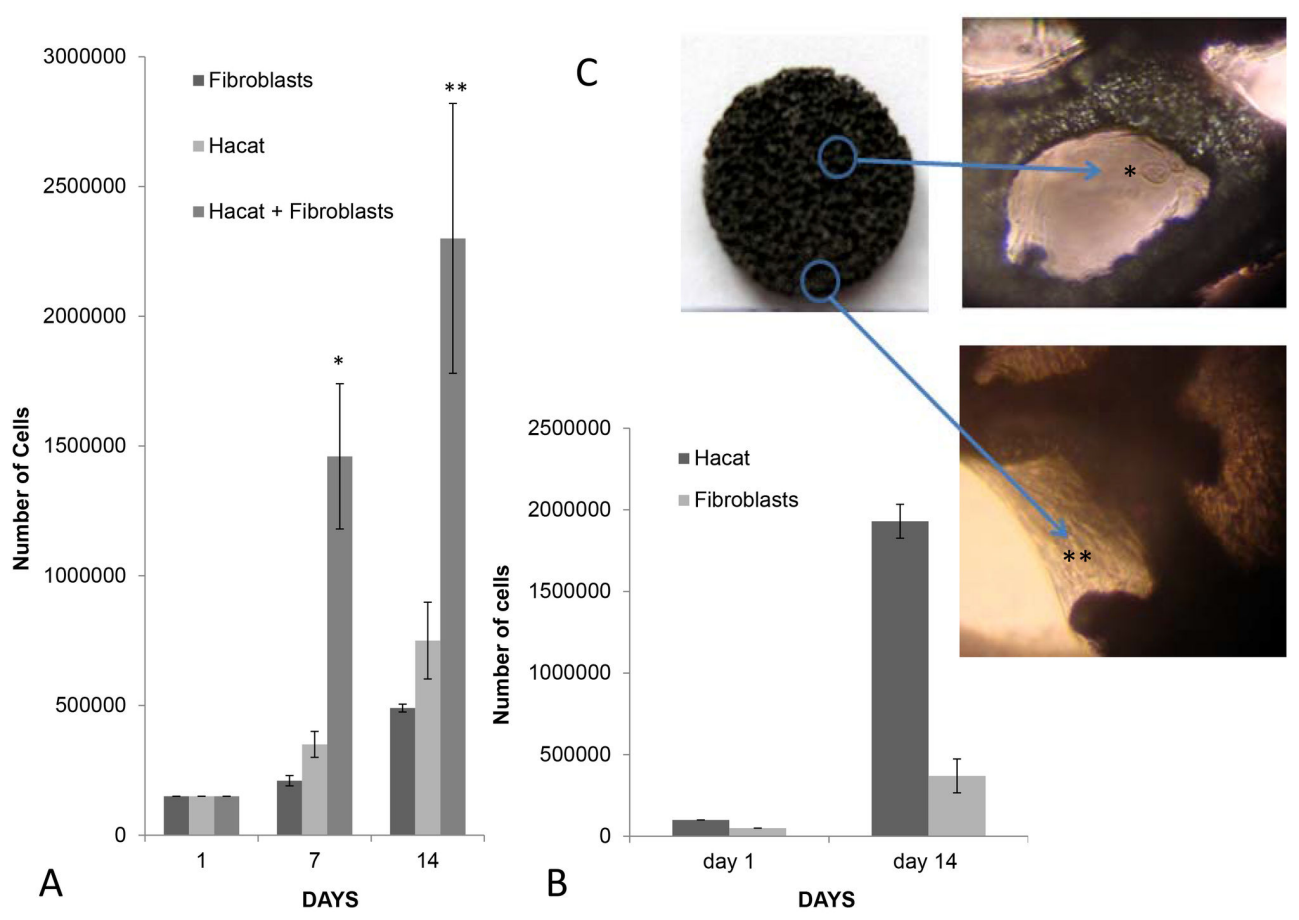
Hacat keratinocytes and fibroblasts growth in the matrices. (A) Growth curves of Hacat keratinocytes and/or fibroblasts cultured in the matrices: The differences between the co-culture and the separated components are all statistically significant (*p < 0.001; **p < 0.001) and the results shown are the average of three different experiments ± standard deviation. (B) At the 14^th^ day of culture the matrices seeded with Hacat keratinocytes and fibroblasts at a 2:1 ratio were predominantly constituted by the former cell type. The cells were distinguished by CD10 expression and the results shown are the average of three different experiments. (C) Images of cells attached either to the matrix borders (*) or inner niches (**). The tantalum skeleton of the matrix appears in black. Light microscope image (100X).

Examination of the attached cells collected from the matrices after 1 or 2 weeks of culture revealed a greater increase in the number of keratinocytes compared with the fibroblasts (Figure 5A) and at 2 weeks the keratinocytes constituted 84 ± 4.5% of total cells (Figure 5B). The cells attached to the scaffold were visible by light microscopy (Figure 5C).

**Figure 6:**
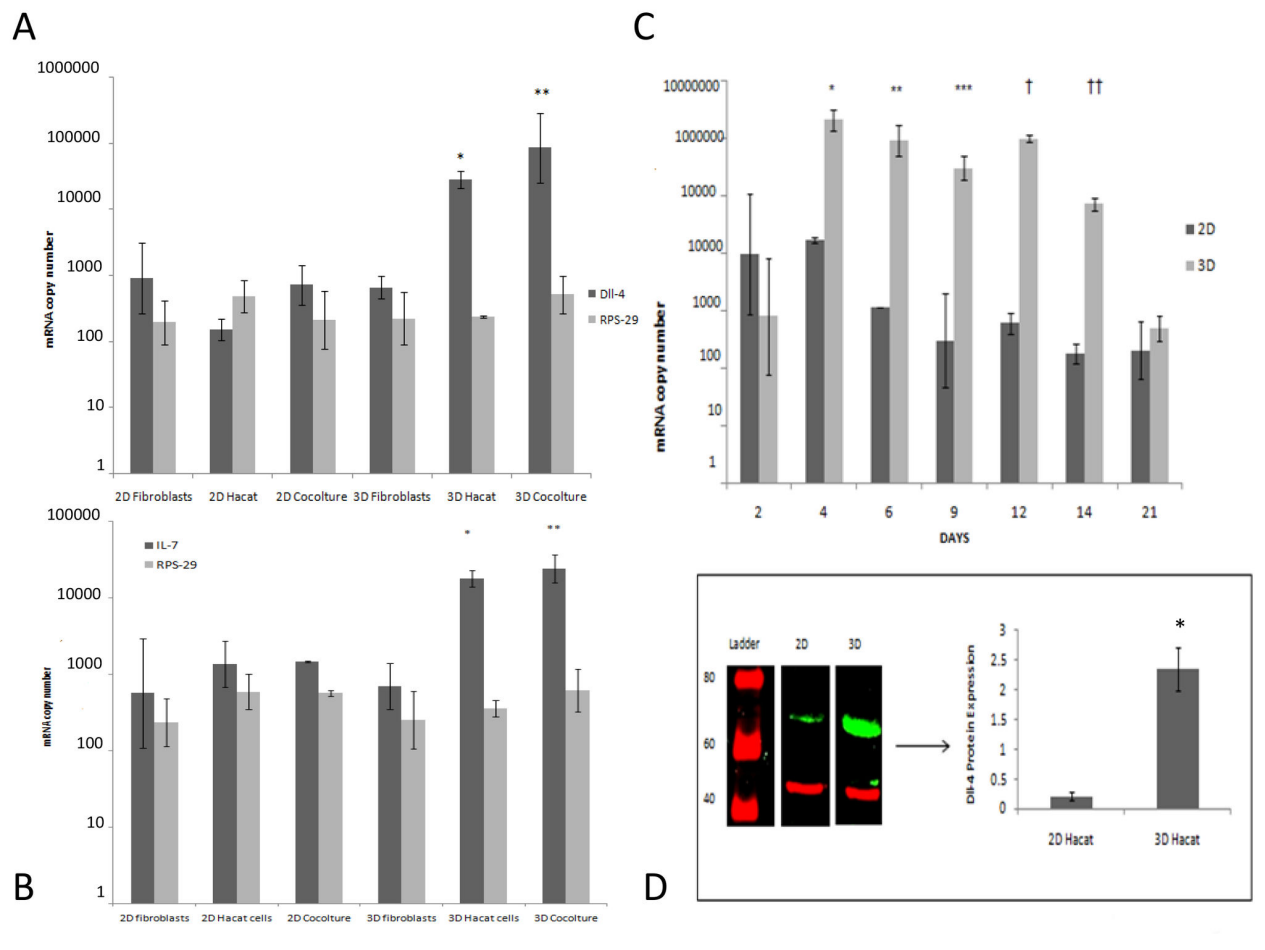
Dll-4 and IL-7 are up-regulated by three-dimensional cultured keratinocytes. (A) Dll-4 gene expression is strongly up-regulated in 3D cultured Hacat keratinocytes either alone or in the presence of fibroblasts. The differences between 3D and 2D either Hacat (*p<0.01) or cocultures (**p<0.05) are significant. No differences were observed in the housekeeping gene expression in all the conditions tested. The results shown are the average of three different experiments ± standard deviation. (B) IL-7 gene expression is strongly up-regulated in 3D cultured Hacat keratinocytes either alone or in the presence of fibroblasts. The differences between 3D and 2D either Hacat (*p<0.05) or co-cultures (**p<0.05) are significant. No differences were observed in the housekeeping gene expression in all the conditions tested. The results shown are the average of three different experiments ± standard deviation. (C) Time dependent up-regulation of the Dll-4 gene in three-dimensional Hacat keratinocytes/ fibroblasts co-cultures. A strong induction is observed during the first week and this high expression is maintained for about 10 days. The results shown are the average of three different experiments ± standard deviation and the differences between 2D and 3D within days 4-14 (* p < 0.01; ** p < 0.01; *** p <0.05; † p < 0.01; † † p < 0.01), are all statistically significant. (D) Dll-4 protein expression in 2D and 3D cultured Hacat keratinocytes. The western blot image shows different parts of one single gel. The average of Dll-4 level of expression normalized to actin from three different experiments ± standard deviation differs between the 2D and 3D environment and the difference is significant (p< 0.001).

Investigation of gene expression by quantitative RT-PCR comparing three dimensional cultures with cells from planar surfaces revealed that the keratinocytes both alone and along with fibroblasts up-regulated Dll-4 expression significantly (p<0.05 n=3) when in the matrix (Figure 6A). In addition under these conditions IL-7 was also found up-regulated (Figure 6B). No change was observed in regard of delta-like ligand 1 (Dll-1) as well as housekeeping RPS-29 genes expression. Time course experiments showed that the highest Dll-4 gene induction occurred within 4-14 days of culture (Figure 6C).

We undertook western blot experiments to determine whether this increase in mRNA of Dll-4 translated into an increase in protein expression in these cells. Our results revealed Dll-4 protein up-regulation in keratinocytes three-dimensional cultures. Dll-4 protein was detected as a 75 kDa band and actin (40 kDa band) was used for normalization (Figure 6D).

### TREC analysis

**Figure 7:**
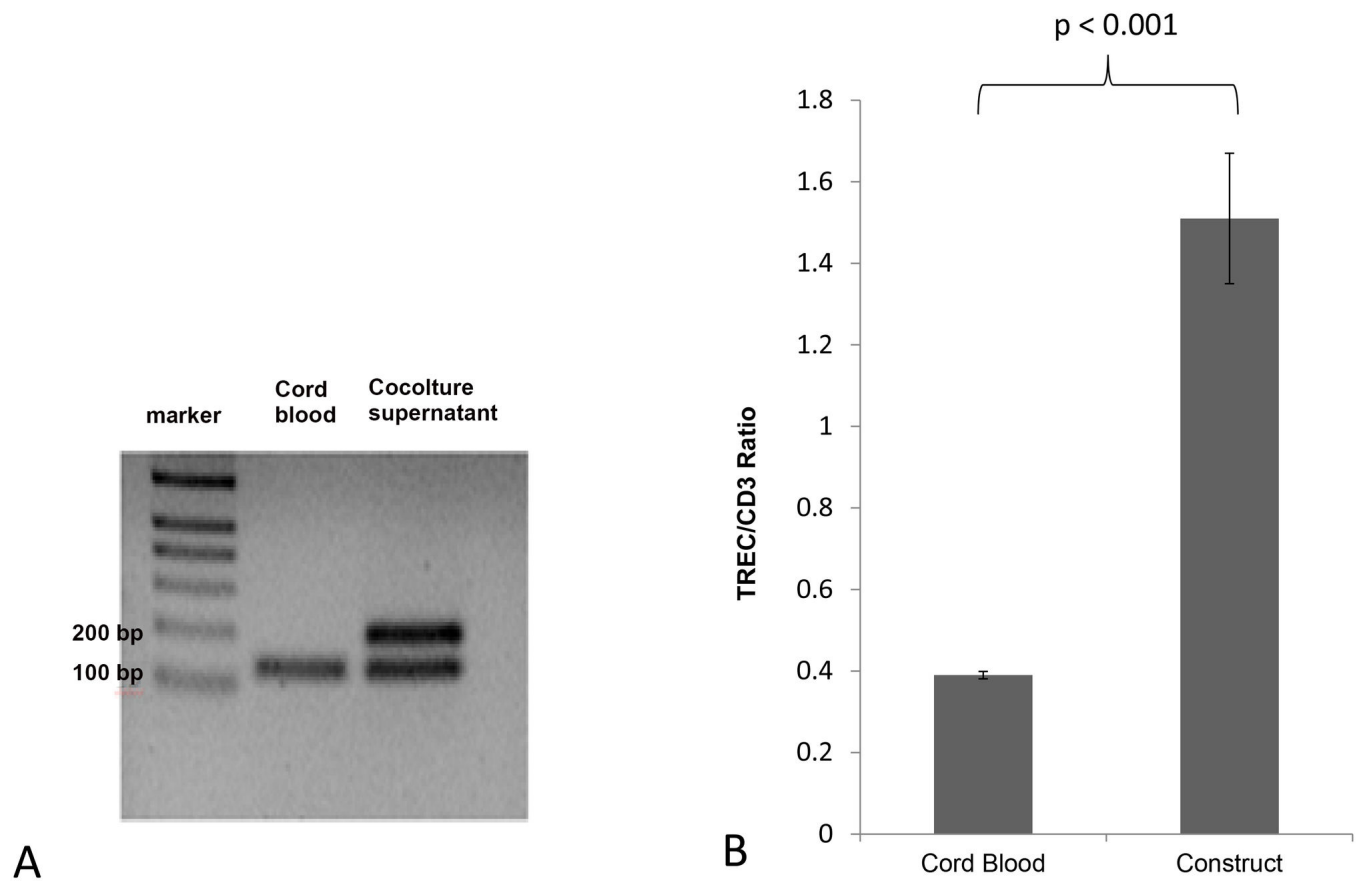
TREC analysis shows that thymocytes were generated de novo. (A) TREC was amplified from DNA from cells generated in the matrices after 10 days of co-culture but not from DNA from cord blood separated precursors. RPS-29 housekeeping gene was amplified in both cases. TREC and RPS-29 were respectively identified as a band of 192 and 142 base pairs. (B) TREC/CD3^+^ ratios from cord blood T cells and thymocytes generated in the matrices. The latter cells show higher level of TREC expression per cell compared to T cells which were separated from cord blood. The results shown are the average of three different experiments± standard deviation and the difference is significant (p < 0.001).

To further confirm the *in vitro* commitment of the progenitors used to the T cell lineage we analysed TREC levels in both an aliquot of cord blood CD34^+^ cells seeded into the skin construct and some CD3⁺ cells generated from these after 10 days of co-culture. Our results from three independent experiments revealed a band corresponding to the TREC amplicon observed only from newly generated T cells and not from the original population of seeding cells. Moreover we quantified TREC levels from both CD3⁺ cells generated from the skin systems and separated from cord blood and the concentration resulted to be 1.51± 0.16 per new generated cell and 0.39± 0.09 per cord peripheral T cell (p < 0.001). These results are shown in Figure 7.

### Cord and peripheral CD34⁺ cells are dissimilar

**Figure 8:**
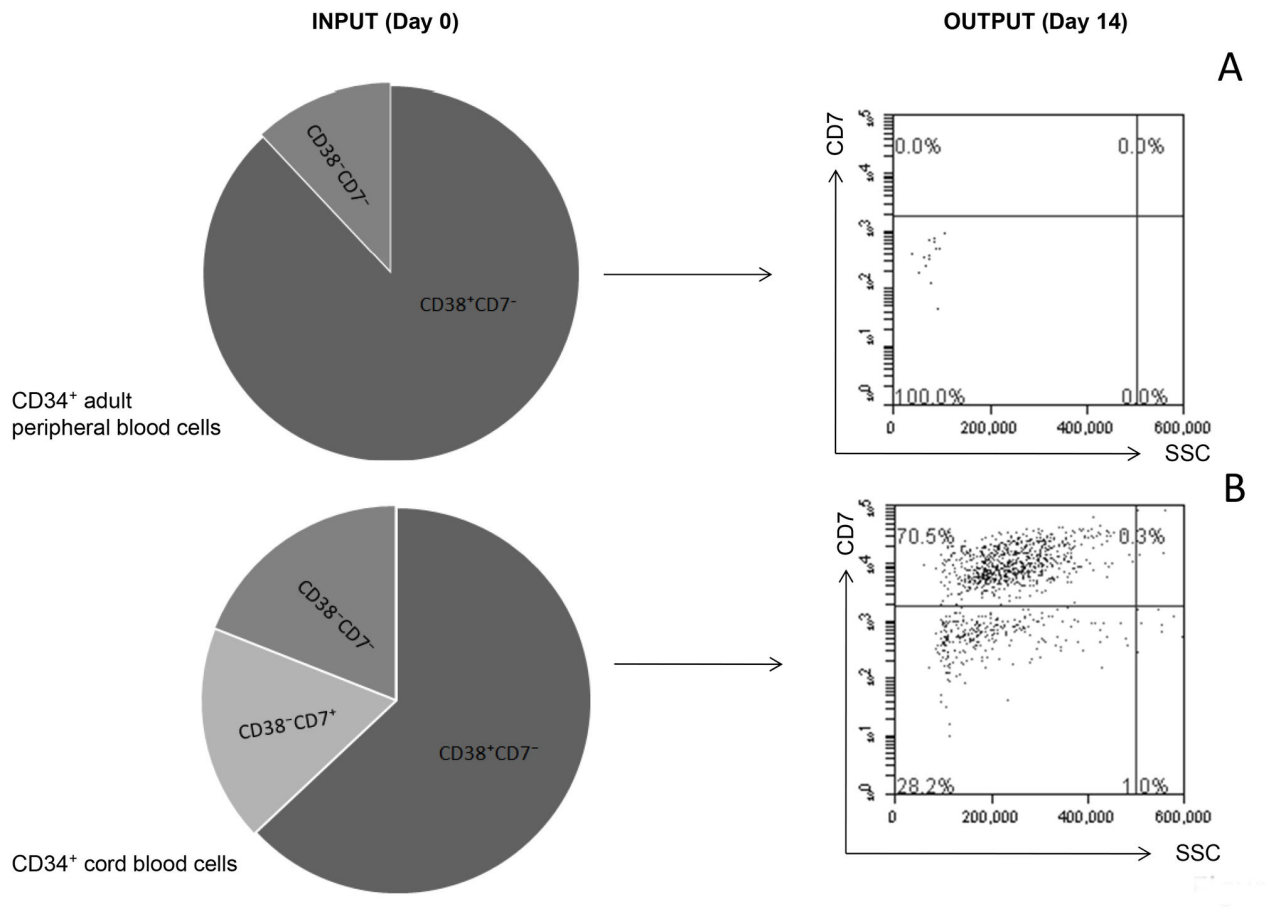
Cord and peripheral CD34⁺ precursors are dissimilar. (A) Thymocytes were never found in the supernatant of matrices seeded with CD34⁺ adult peripheral blood cells and checked up to 2 weeks of co-culture. (B) Cord blood cells gave rise to CD7^+^thymocytes. The images are representative of three different experiments all performed in parallel and reports initial differences in the CD34⁺ cells subsets composition.

Three different experiments were performed comparing the same number of CD34⁺ cells separated from either adult peripheral or cord blood and cultured in the three dimensional matrix system. In all the conditions tested the adult CD34+ cells died within the first 72 hours of culture whereas the cord blood progenitors actively proliferated and generated thymocytes. All the cultures were maintained for up to two weeks. Our phenotypic analysis of the cells from either source showed distinct differences. In adult blood 88 ± 4 % CD34⁺ cells were CD38⁺ versus 63 ± 3 % in cord blood (p < 0.001). Furthermore 18 ± 0.7% of cord blood CD34⁺ cells were CD7⁺ whereas no CD34⁺CD7⁺ cells were detected in adult blood. These results are shown in figure 8.

## Discussion

The work reported here shows that commitment and development of cord blood stem/progenitor cells into cells with the phenotype of mature thymocytes was achieved by placing them within a three-dimensional tantalum coated matrix coated with fibroblasts and keratinocytes in media containing IL-7, IL-15, and Flt-3L. The human T cell developmental pathway in the thymus has been defined phenotypically with stages including CD34^+^CD7^-^CD1a^-^ cells giving rise to pre-T cells which are with CD34^+/lo^CD7^+^CD1a^+^ which develop through an immature single positive stage of CD7^+^CD1a^+^CD4^+^ to double positive cells CD3^+^CD4^+^CD8^+^ to produce single positive cells either CD3^+^CD4^+^CD8^-^ or CD3^+^CD4^-^CD8^+^ [16].

Both phenotypic and genotypic analysis revealed that differentiation was ordered and progressed along the normal developmental pathway as judged by the sequential appearance of the expected intermediate stages in the production of mature thymocytes [15–19]. This system is probably not as efficient as the fetal thymic organ culture system where a single progenitor can be induced to develop into CD4 or CD8 cells [20], but we were able to see differentiation from a few hundred CD34^+^ cells and further refinement may lead to better efficiency. However in the absence of a three-dimensional cellular environment we were unable to observe any T cells development from progenitor cells. We therefore consider the critical factors in this work to be the three-dimensional aspects of the culture, the coating stromal cell equivalents and the source of the progenitor cells used.

The requirement for three dimensional cultures systems for inducing T cell development was first shown for murine systems [21] and this was recently linked to the expression of the notch ligands which were expressed highly by stromal cells cultured in three dimensions but down-regulated when these cells were cultured as a monolayer [22]. Two dimensional planar cultures can permit the production of a few CD8⁺ T cells from cord blood CD34^+^ precursors after 2 months of culture in the OP9-Dll1 system [23], but our results would suggest that for the efficient development of T cells there would appear to be a central role for a three-dimensional environment in addition to Dll-4 up-regulation.

Both the type and quantity of the Notch ligand plays a pivotal role in inducing and directing the differentiation of progenitor cells towards distinct lympho-haematopoietic lineages [24,25] and this is especially true for Dll-4 [26,27] whose role in T cell development is essential [28–30]. In our study up-regulation of Dll-4 was not immediately seen after placing the keratinocytes on the tantalum matrix but was delayed by some days, suggesting that the three dimensional orientation was only one of the factors involved. A secondary factor may be cell confluence as up-regulation of Dll-4 increased during the culture period to peak at 4 days which we believe was associated with the cells becoming confluent within the matrix. No major up-regulation of Dll-4 differentiation was apparent when these cells were undergoing density dependant inhibition in a planar position. Occasionally we have noted that the keratinocytes in our matrix cultures either failed to show Dll-4 up-regulation or only showed limited increases. In the former case CD34^+^ cells were unable to produce T cells and in the latter case we noted that the frequency of T cells produced was much lower than expected. From this we suggest that others may have had the problem of failure in up-regulation of Dll-4 and as a consequence may have failed to generate T cells. This could account for the difficulty expressed by others [9] in trying to repeat the work of setting up thymus equivalent cultures using skin derived cells [7].

One concern we had was the amplification of passenger T cells in our system. We feel that we can confidently exclude this. First we found no evidence of T cells either by phenotype or genotype (TREC) analysis in the purified CD34^+^ population used for seeding the matrix. Secondly our sjTREC analysis of cells generated in matrix cultures revealed values of about 1.5 sjTREC per cell which can only be explained by the generation of cells which had just rearranged their TCR but had not undergone significant proliferation after rearrangement. This value fits with calculations based on previous evidence that sj-TREC was observed in about 70% of all newly generated αβ T cells [31] and that only a maximum of two sjTRECs are present in any newly generated αβ T cells [32]. Finally we observed the sequential expression of surface markers typically present in thymocytes but not mature T cells such as CD1a, CD38 and CD4 CD8 co-expression, all of which are features of T cell de novo generation [15–19]. This evidence is the basis of our conviction that the CD34^+^ cells were generating cells of the T lineage within the model system. Our inability to generate T cells from adult CD34^+^ cells would suggest differences in the population defined by this marker in adults compared with those from cord blood though less differentiated CD133^+^ bone marrow derived adult cells could still generate T cells [7]. Previous work has shown that cord blood progenitor cells possess extremely high T cell fate potentiality [33] and a progressive loss of this capability and myeloid skewing has been described in precursors from older individuals [34–37]. In our work we tested CD34^+^ non mobilized circulating peripheral blood cells from a 55 years old individual. Failure of haematopoietic progenitors from older individuals to proliferate and differentiate in a specific supportive environment supports limited previous observations that the proliferative potential of human haematopoietic progenitors declines with age [38] and that bone marrow from older humans is less efficient at reconstituting recipients when compared to the reconstitution capacity of bone marrow derived from younger patients [39].

Moreover cord blood progenitors possess a higher capability to differentiate along the T-lineage pathway compared to their adult counterparts [33]. This may be because the CD34^+^ cells from cord blood contain CD7⁺ lympho-committed precursors [40], which may be present in limited amounts in peripheral blood.

Our results suggest that in older individuals the CD34^+^ population may contain only a very limited number of cells with the ability to generate T cells which may be retained in the bone marrow and only exceptionally be released into the periphery. In agreement with this, studies done with adult patients after cancer treatments or bone marrow transplant have shown that T cell generation was derived from expansion of mature peripheral T cells and not T cell de novo generation [41].

Positive and negative selection of the cells generated in these matrices was not a feature of these experiments partly because of the problems with the need to haplotype all of the donor samples and match with the cell lines. This is something we hold in common with those using xenogeneic systems [4,5]. Our aim was to define the simplest model system in order to define critical attributes of the system so that we could more easily construct a thymus using autologous derived progenitors and stromal cells.

This is the first identification that a permissive environment could be synthesised from epithelial and fibroblast cell lines anchored to a three-dimensional matrix in a media containing growth factors. This study reveals that the critical elements of the environment were the presence of sufficient quantities of the Notch ligand Dll-4 and a three dimensional matrix. This study links these two elements showing that positioning of an epithelial cell line within the three dimensional matrix leads to the significant up-regulation of Dll-4. In this system we show that whilst differentiation of cord blood derived CD34^+^ cells was efficient this was not the case with the CD34^+^ population from adult blood where no clear T cell differentiation was apparent.
